# Exploring metabolic pathway disruption in the subchronic phencyclidine model of schizophrenia with the Generalized Singular Value Decomposition

**DOI:** 10.1186/1752-0509-5-72

**Published:** 2011-05-16

**Authors:** Xiaolin Xiao, Neil Dawson, Lynsey MacIntyre, Brian J Morris, Judith A Pratt, David G Watson, Desmond J Higham

**Affiliations:** 1 Department of Mathematics and Statistics, University of Strathclyde, Glasgow, G1 1XH, Scotland, UK; 2 Psychiatric Research Institute of Neuroscience in Glasgow (PsyRING), Universities of Glasgow and Strathclyde, G12 8QQ, UK; 3 Strathclyde Institute of Pharmacy and Biomedical Sciences (SIPBS), University of Strathclyde, Glasgow, G4 0NR, UK; 4 Center for Neuroscience, University of Strathclyde (CeNsUS), Glasgow, G4 0NR, UK; 5 Institute of Neuroscience and Psychology, College of Medical, Veterinary and Life Sciences, University of Glasgow, Glasgow, G12 8QQ, UK

## Abstract

**Background:**

The quantification of experimentally-induced alterations in biological pathways remains a major challenge in systems biology. One example of this is the quantitative characterization of alterations in defined, established metabolic pathways from complex metabolomic data. At present, the disruption of a given metabolic pathway is inferred from metabolomic data by observing an alteration in the level of one or more individual metabolites present within that pathway. Not only is this approach open to subjectivity, as metabolites participate in multiple pathways, but it also ignores useful information available through the pairwise correlations between metabolites. This extra information may be incorporated using a higher-level approach that looks for alterations between a pair of correlation networks. In this way experimentally-induced alterations in metabolic pathways can be quantitatively defined by characterizing group differences in metabolite clustering. Taking this approach increases the objectivity of interpreting alterations in metabolic pathways from metabolomic data.

**Results:**

We present and justify a new technique for comparing pairs of networks--in our case these networks are based on the same set of nodes and there are two distinct types of weighted edges. The algorithm is based on the Generalized Singular Value Decomposition (GSVD), which may be regarded as an extension of Principle Components Analysis to the case of two data sets. We show how the GSVD can be interpreted as a technique for reordering the two networks in order to reveal clusters that are exclusive to only one. Here we apply this algorithm to a new set of metabolomic data from the prefrontal cortex (PFC) of a translational model relevant to schizophrenia, rats treated subchronically with the N-methyl-D-Aspartic acid (NMDA) receptor antagonist phencyclidine (PCP). This provides us with a means to quantify which predefined metabolic pathways (Kyoto Encyclopedia of Genes and Genomes (KEGG) metabolite pathway database) were altered in the PFC of PCP-treated rats. Several significant changes were discovered, notably: 1) neuroactive ligands active at glutamate and GABA receptors are disrupted in the PFC of PCP-treated animals, 2) glutamate dysfunction in these animals was not limited to compromised glutamatergic neurotransmission but also involves the disruption of metabolic pathways linked to glutamate; and 3) a specific series of purine reactions Xanthine ← Hypoxyanthine ↔ Inosine ← IMP → adenylosuccinate is also disrupted in the PFC of PCP-treated animals.

**Conclusions:**

Network reordering via the GSVD provides a means to discover statistically validated differences in clustering between a pair of networks. In practice this analytical approach, when applied to metabolomic data, allows us to quantify the alterations in metabolic pathways between two experimental groups. With this new computational technique we identified metabolic pathway alterations that are consistent with known results. Furthermore, we discovered disruption in a novel series of purine reactions that may contribute to the PFC dysfunction and cognitive deficits seen in schizophrenia.

## Background

### Background in neuroscience and metabolomics

Schizophrenia is characterized by deficits in cognition known to be dependent upon the functional integrity of the prefrontal cortex (PFC). Furthermore, compromised PFC function in schizophrenia is supported by a multitude of neuroimaging studies reporting hypometabolism ('hypofrontality'), as evidenced by decreased blood flow or glucose utilization [1,2]. While the pathophysiological basis of PFC dysfunction in schizophrenia is not completely understood, a central role for NMDA receptor hypofunction is widely supported. For example, subchronic exposure to the NMDA receptor antagonist phencyclidine (PCP) induces cognitive deficits and a 'hypofrontality' which directly parallels that seen in schizophrenia [3-5]. Furthermore, subchronic PCP exposure induces alterations in GABAergic cell markers and 5-HT receptor expression in the PFC similar to those seen in this disorder [3,6,7]. While this evidence places NMDA receptor hypofunction central to the pathophysiology of PFC dysfunction in schizophrenia, the mechanisms through which NMDA hypofunction promotes PFC dysfunction are poorly understood.

Metabolomics is the comprehensive analysis of small molecule metabolites in biological systems [8]. It involves the study of the metabolome which is defined as all of the small molecular weight compounds within a sample that are required for metabolism, whose roles include growth and functionality [9-11]. Sample sources include bacteria, parasites, animals and humans and sample types can include biofluids, cells or tissue extracts. Metabolomics can be utilized as a tool for the characterization and quantification of all of the metabolites in a biological system. Its applications include profiling disease biomarkers [12,13], monitoring disease progression [14], investigating xenobiotic metabolism [15], investigating drug-induced toxicity [16,17] and investigating metabolism in genetically modified animals [18]. Mass spectrometry (MS) has been employed extensively as an analytical platform for metabolomics studies [19-21]. The popularity of this approach has increased over the last decade, in part due to the advent of high resolution Fourier transform mass spectrometers which offer improved reproducibility, accuracy and sensitivity. This makes mass spectrometry suitable for high throughput metabolomics studies [22]. In addition, the Orbitrap mass spectrometers that are now available offer similar performance to FT-MS systems without the need for a high strength magnetic field [23]. HILIC chromatography has been utilized as a separation technique prior to MS detection of polar metabolites in aqueous biofluids such as urine, serum and plasma [24-30].

Additionally, it has also been used for the detection of multiple neurotransmitters in primate cerebral cortex [31]. HILIC chromatography has been chosen for metabolomic studies as it is useful for the analysis of highly polar metabolites which are poorly retained on reverse phase columns [32]. Detailed reviews of the principles and applications of HILIC have been previously outlined [25,33]. Here, HILIC-chromatography is utilized in combination with an LTQ-Orbitrap for metabolic profiling of metabolite extracts from the PFC of control and PCP-treated rats.

Metabolomics represents a robust approach through which alterations in diverse metabolic pathways may be determined at a biological systems level. In this way a metabolomics approach may prove useful in further elucidating the pathophysiological mechanisms contributing to PFC dysfunction in schizophrenia. Furthermore, this approach may also allow for the identification of PFC metabolic biomarkers for the cognitive deficits in this disorder. While the metabolomics approach can provide a rich and comprehensive set of data, the appropriate quantitative analysis of this data has not been adequately developed. In particular, the identification of statistical differences in metabolic pathways between experimental groups rather than the identification of statistical differences in individual metabolites alone represents a major challenge to quantitatively identifying metabolic alterations at a systems level from metabolomic data. One method through which statistical differences in metabolic pathways can be identified from metabolomic data involves the representation of this data as a large, complex network of nodes (single metabolites) connected by real-value edges (the correlation coefficient between two metabolites). This form of representation has high face validity as the relationship between two metabolites, in a given pathway, is governed by a single or series of enzymatic reactions that can be viewed as being represented by the correlation between the concentrations of the two metabolites. Another advantage is that metabolomic data consist of a range of metabolites detected in both of the experimental groups of interest meaning that these data can be expressed as two complex networks based upon the same set of nodes. This data structure is amenable to analysis through the application of the Generalized Singular Value Decomposition (GSVD) algorithm.

### Background in network science and spectral methods

Large, complex interaction networks arise across many applications in science and technology [34-36]. Spectral methods, based on information computed from eigenvectors or singular vectors, have been used successfully to reveal fundamental network properties. For example, we may wish to cluster objects into groups [37], put objects into order [38] or discover specific patterns of connectivity within subgroups [39-42]. In this work, we look at the case where two interaction data sets are available and the aim is discover differences between the two sets in the form of mutually exclusive clusters. For example, a given group of biologically defined entities, such as genes, proteins, metabolites or brain regions, may contain a subgroup that behaves in a coordinated manner under one condition, or in one organism, but not in another--the network with respect to one type of interaction contains a cluster that is not present in the other. We will show that the Generalized Singular Value Decomposition, which is becoming more widely used in computational biology [43,44] can be justified as the basis of a network reordering approach. We also consider how to quantify the statistical significance of network patterns that are uncovered.

Overall, this work develops and applies a novel algorithm in network science and shows that it reveals meaningful insights when applied to cutting-edge metabolomic data.

## Results

### Derivation of new algorithm

Suppose that the square, symmetric, real-valued matrices *A *and *B *in ℝ^*N*×*N *^represent two different types of interaction between a set of *N *nodes. We have in mind the case where the weights play the role of correlation coefficients. Our aim is to discover clusters, in the sense of subsets of nodes that are mutually, pairwise, strongly connected through positive weights. The algorithm will also discover clusters of strong negative connectivity, although in practice this type of pattern is less likely to be present. However, we note that the arguments given below and the resulting algorithm remain valid in the case where the weights are non-negative, with zero representing the minimal level of similarity. The novelty of our approach is that in order to reveal interesting differences between the two types of connectivity data, we look for a set of nodes that form a good cluster with respect to *A *and a poor cluster with respect to *B*, or vice versa. As a starting point for a computational algorithm, we consider the identity(1)

for *x *∈ ℝ*^N^*. Here ||·||_2 _denotes the Euclidean norm and  is one way to generalize the concept of out-degree to the case of a weighted network. Suppose we wish to split the nodes into two groups such that nodes within each group are well-connected but nodes across different groups are poorly connected. We could use an indicator vector *x *∈ ℝ*^N ^*to denote such a partition, with *x_s _*= 1 if node s is placed in group 1 and *x_s _*= -1 if node *s *is placed in group 2.

Fixing on two nodes, *k *and *l*, we could argue that the existence of a third node, *i*, such that *a_ik _*and *a_il _*are both large and positive or both large and negative is evidence in favor of placing *k *and *l *in the same group (since they have in common a strong similarity or dissimilarity with node *i*). On the other hand small or oppositely signed values for *a_ik _*and *a_il _*is evidence in favor of placing *k *and *l *in different groups. In terms of the indicator vector, this translates to

**1**. *a_ik_a_il _*large and positive ⇒ try to choose *x_k_x_l _*= +1,

**2**. *a_ik_a_il _*small or negative ⇒ try to choose *x_k_x_l _*= -1.

Returning to the right-hand side of (1), we see that  is independent of the choice of indicator vector, and  gives a measure of how successfully we have incorporated the (possibly conflicting) desiderata in points 1 and 2 over all pairs *k*, *l *and third parties *i*. So we could judge the quality of an indicator vector by its ability to produce a large value of , provided other constraints, such as balanced group sizes, were satisfied.

Analogously, we can argue that making  as negative as possible is a good way to avoid forming well-connected subgroups, and so the problem(2)

is a good basis for picking out strong clusters in *A *that are not present in *B*.

In general, optimizing over a large, discrete set of possibilities is computationally intractable, and hence we will follow the widely used practice of relaxing to an optimization over ℝ*^N^*[37,45]. This approach goes back as far as the pioneering work of Fiedler [46] and has some theoretical underpinning in the case where a single network is analyzed [47,48]. So, instead of (2) we have(3)

At this stage we recall that a general pair of matrices *A *∈ ℝ^*m*×*n *^and *B *∈ ℝ^*p*×*n *^can be simultaneously factorized using the Generalized Singular Value Decomposition (GSVD) into(4)

where *U *∈ ℝ^*m*×*m *^and *V *∈ ℝ^*p*×*p *^are both orthogonal, *X *∈ ℝ^*n*×*n *^is invertible, *C *∈ ℝ^*m*×*n *^and *S *∈ ℝ^*p*×*n *^are diagonal with nonnegative entries such that *C *= diag(*c*_1_, *c*_2_,..,*c_n_*) and *S *= diag(*s*_1_, *s*_2_,..., *s_q_*) with *q *= min(*p*, *n*), and 0 ≤ *c*_1 _≤ *c*_2 _≤ ··· ≤ *c_n _*and *s*_1 _≥ *s*_2 _≥ ··· ≥ *s_q _*≥ 0 [49]. The ratios *λ_i _*= *c_i_*/*s_i _*are the *generalized singular values *of *A *and *B*.

A key property of the GSVD is that the columns of *X *are stationary points of the function f :ℝ*^n ^*↦ ℝ given by *f*(*x*) = || *Ax *||_2 _/||*Bx *||_2_, with the generalized singular values *λ_i _*giving the corresponding stationary values. Hence, we may tackle the problem (3) through the GSVD. Columns 1, 2, 3,... of *X *are candidates for finding good clusters in *B *that are poor clusters in *A *and, analogously, columns *N*, *N *- 1, *N *- 2,... of *X *are candidates for finding good clusters in *A *that are poor clusters in *B*.

To transform back from real to discrete domains, we may use the ordering of the elements in *x *to define a new ordering for the two networks. More precisely, we relabel row and column *i *of *A *and *B *as row and column *p_i_*, where

In this way, the existence or lack of clusters in each network becomes apparent from inspection of the heat map of the reordered matrix. This is the approach that we use. We will also show that *p*-values can be computed to quantify the statistical significance of the results. The issue of fully automating the choice of cluster size is left as future work.

### A variant of the algorithm

In our context, the matrices *A *and *B *are square, with *m *= *n *= *p *= *N*. In this case, if we make the additional assumption that *A *and *B *are invertible it is known that the GSVD is closely related to the standard Singular Value Decompositions (SVD) of *AB*^-1 ^and *BA*^-1^. To see this, we could rearrange (4) into(5)

Alternatively, we may let *z *= *Ax *or *y *= *Bx *in (3), to obtain the quadratic problems

which can be solved through the standard SVD.

It is known from spectral graph theory that the dominant singular vectors give good directions in which to look for clusters [37,50]. Inverting the weight matrix reverses their importance (the singular value*σ *becomes *σ *^-1^) and hence a spectral clustering approach applied to *A*^-1 ^will typically find the opposite of good clusters--poorly connected nodes will be grouped together [51]. So, intuitively, forming *AB*^-1 ^in (5) should produce a data matrix for which the SVD approach finds good clusters for *A *and poor clusters for *B*. Analogously, the opposite holds for *BA*^-1^.

Having interpreted the algorithm this way, it is then natural to consider the reverse products, *A*^-1^*B *and *B*^-1^*A*, or, equivalently, to form the optimization problem(6)

We may interpret (6) from the point of view that making *B*^-1^*x *large encourages poor clusters for *B*, while making *A*^-1^*x *small encourages good clusters for *A*. In this case, we would base our algorithm on the GSVD of *A*^-1 ^and *B*^-1^.

In the situation where *A *and *B *are both symmetric, corresponding to undirected networks, we have, from (4),

and

Then we may appeal to the arguments given previously and use columns from the inverse of the third factor in the GSVD as the basis for reordering. With this approach we use columns of *X *^-*T *^rather than columns of *X*. We emphasize that although this heuristic derivation used an assumption that *A *and *B *are invertible, the GSVD, and hence the final algorithm, applies in the non-invertible case. Also, the algorithms that we use do not require the computation of matrix inverses.

In tests on both synthetic and real network pairs, we found that this version of the algorithm was more effective, [52]. Hence, in this work we focus on the approach of reordering networks pairs via columns of *X *^-*T*^. In summary, the first few columns of *X *^-*T *^should give orderings that favor clusters in *B *rather than *A *and vice versa for the final few columns. In our computational examples, we used the gsvd routine built in to MATLAB http://www.mathworks.com/.

### Synthetic test on binary networks

**Figure 1:**
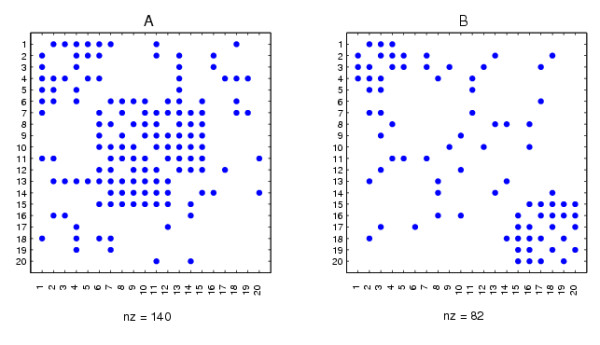
**Adjacency matrices for the two synthetic networks**.

In this section we illustrate the algorithm in a simple, controlled case where we know the "correct" answer. We begin by considering binary networks, where results can be clearly visualized. We generated binary adjacency matrices *A *and *B *as shown in Figure 1. Here we have 20 nodes. In both networks, nodes 1-5 are well connected. In *A *there is a well connected cluster consisting of nodes 6-15, whereas in *B *there is a well connected cluster consisting of nodes 15-20. To make the test more realistic, the clusters are not perfect; there are both missing edges (false negatives) within the clusters and spurious edges (false positives) outside the clusters. Our aim is to test whether the algorithms can identify the clusters that are particular to each data set. We then show how statistical significance can be quantified.

**Figure 2:**
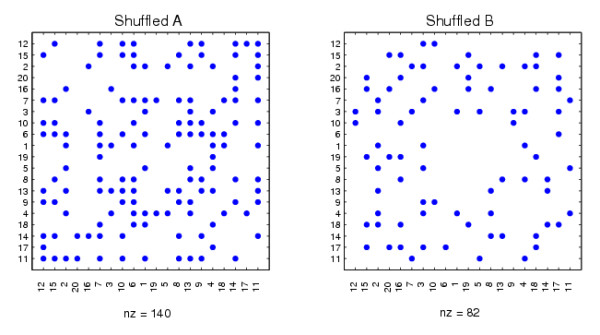
**Relabeled versions of the synthetic networks in Figure 1**.

We emphasize that the node labeling in Figure 1 was chosen purely to make the inherent structure visually apparent. Any spectral reordering algorithm should be invariant to a relabeling of the input data. In our context, this follows from the fact that for any permutation matrix *P*, the factorizations *A *= *UCX *^-1 ^and *B *= *V SX *^-1 ^are equivalent to *PAP^T ^*= (*PU*)*C*(*PX*) ^-1 ^and *PBP^T ^*= (*PU*)*S*(*PX*) ^-1^. So, on the relabeled data matrices, (*PX*) plays the role that was played by *X*, and our algorithm reorders based on the appropriately permuted columns of *X *^-*T*^, as required. In Figure 2 we show the same two data sets with an arbitrary relabeling in order to illustrate that the inherent structure is no longer apparent. In essence, we are hoping that the algorithm will find the structure that has been buried in Figure 2.

**Figure 3:**
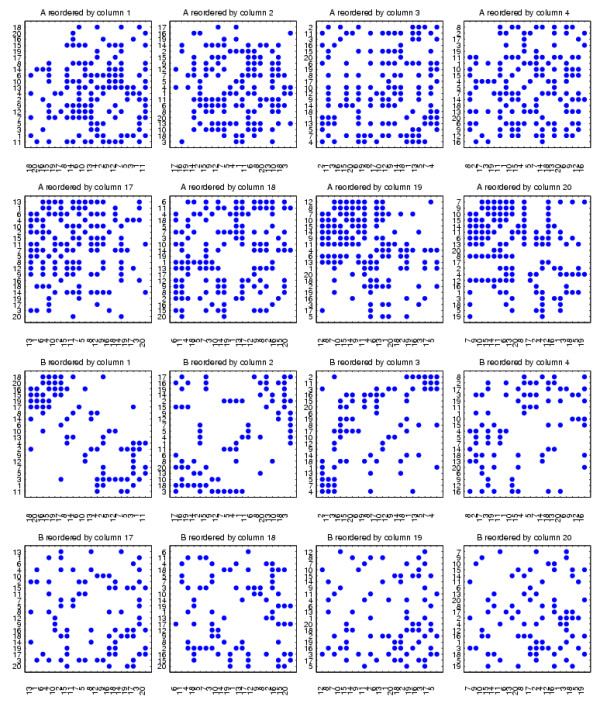
**Networks reordered using columns of ***X ***^-***T***^**.

In Figure 3 we display the two adjacency matrices reordered with the algorithm; we show reordering with eight different columns of *X *^-*T*^, four from each end of the spectrum. We see that mutually exclusive structures have been uncovered. The reordering from the first column begins with nodes 18, 20, 16, 15, 19, 17, which form a cluster in *B*, but not *A*. The final column begins by picking out nodes 7, 9, 10, 15, 14, 11, 6, 13, which form the bulk of the 6-15 cluster in *A*. Nodes 8 and 12, which are missing from this sequential ordering, are placed at the head of the ordering in the penultimate column, which begins 12, 8, 7, 10, 15, 14, 9, 11. So in summary, the 19th and 20th columns of *X *^-*T *^each reveal almost complete information about the exclusive cluster in *A*, and between them they capture the full cluster.

### Cluster validation

Suppose we find *τ *nodes giving a good cluster *s *for *B *but a poor cluster for *A *when the graphs are reordered by column *v *from *X *^-*T*^. Is this type of substructure likely to arise "by chance"? The following general approach can be used in order to determine a *p *-value, where we will regard a value below 0.05 as indicating statistical significance.

Initialization: Compute a measure of cluster quality, *c*(*A*, *B*), for the promising substructure consisting of those *τ *nodes in networks *A *and *B *reordered by column *v*.

Step 1: Randomize the networks and obtain new data sets  and .

Step 2: Compute the GSVD for the randomized networks  and  and obtain a matrix .

Step 3: Compute the measure  for the *τ *node 'cluster' in  and  reordered by column *v *from .

*p *-value After performing *M *loops over Steps 1 to 3, compute a *p *-value as the proportion of  samples that exceed *c*(*A*, *B*).

For our cluster quality measure *c*(*A*, *B*) we used

For these binary graphs, the density *f *(*s*) of cluster *s *was defined as(7)

Here, |*E*(*s*)| represents the actual number of edges in the object block *s*, and |*s*| is the maximum possible number of edges.

For weighted graphs, in the case where the cluster is dominated by positive weights, we will generalize this to(8)

Here, *w*(*s*) denotes the average weight in block *s*. We note the denominator |*s*| cancels when ratios are computed in the *p*-value algorithm.

In Figure 3, we see that eight nodes 7,9,10,15,14,11,6,13 form a cluster in *A*, but not in *B*, when the synthetic data is reordered with the final column of *X *^-*T*^. Applying the procedure above, using permutation to randomize the networks *M *= 1000 times as described below, we obtained a *p*-value of 0.007. Applying the same procedure, we also obtained a *p*-value of 0.029 for the first 6 nodes 18, 20, 16, 15, 19, 17 when the synthetic data is reordered with the first column of *X *^-*T*^, which visually form a cluster in *B*, but not in *A*. These *p*-values (< 0.05) both indicate that the results are statistically significant. As a further test, we arbitrarily selected the subnetworks of *A *and *B *composed of nodes 2,4,12,16,1,3,18, which correspond to the 12th to 18th components of the sorted final column from *X *^-*T*^. In this case, we would not expect to find a significant result. This is reflected in the large *p*-value of 0.844. In more exhaustive experiments, three randomization methods were tested [52]:

**• Erdös-Rényi: **generate a classical random graph with the appropriate number of edges.

**• Redistribution: **redistribute the entries in each row and each column of *A*, and perform the same operations on *B*.

**• Permutation: **reorder the nodes in *A *and *B *and choose the first *τ *nodes in this new ordering. In this case, recomputation of the GSVD in Step 2 is not necessary, due to the permutation invariance of the factorization.

Of those three approaches, Erdös-Rényi may be the most commonly used method to randomize a binary network, whereas permutation extends most naturally to the case of weighted edges, so we used permutation in the test shown here. We also tested another simple cluster quality measure which is the ratio of density of edges within the cluster in one graph and that in the other graph.

These variations were studied within this general methodology on both real and synthetic data sets [52]. In all cases, comparable *p *-values were produced.

### Synthetic test on correlation networks

Having tested the algorithm on binary networks, we now consider the case where weighted edges arise as correlation coefficients.

**Figure 4:**
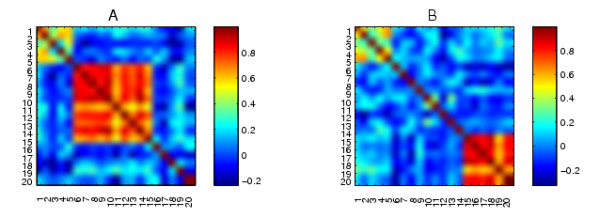
**The original synthetic correlation data**.

First, we generate two correlation matrices *A *and *B *as shown in Figure 4. Here, each graph has 20 nodes, and each entry is real valued, representing the correlation coefficient between the corresponding nodes. The same cluster patterns given for the synthetic binary matrices in Figure 1 were built in to the synthetic correlation data: nodes 1-5 are well connected in both networks; in *A *there is a well connected cluster consisting of nodes 6-15, whereas in *B *there is a well connected cluster consisting of nodes 15-20. Some noise was added to the clusters to make this test more realistic.

**Figure 5:**
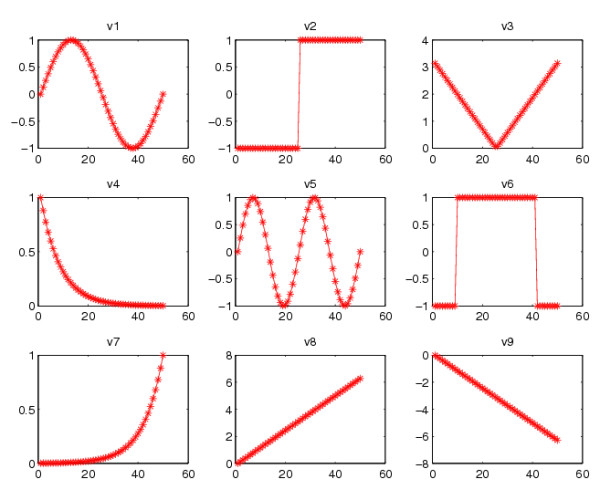
**The nine signals**.

More precisely, in our computation, the value of each entry (the correlation coefficient) in *A *and *B *as shown in Figure 4 is generated from a pair of 20 × 50 rectangular matrices *D_a _*and *D_b_*. The corresponding cluster patterns are built from signals. Figure 5 shows the nine signals that take part in the data. These are row vectors with 50 elements. We use *v*^[1]^, *v*^[2]^, *v*^[3]^,..., *v*^[9] ^to denote them. From these signals, we set up two matrices

• *D_a _*∈ ℝ^20×50^: the first 5 rows are linear combinations of *v*^[1]^, *v*^[2]^, *v*^[3]^, *v*^[4]^, *v*^[5]^, *v*^[6] ^and *v*^[7]^. Rows 6 to 15 are combinations of *v*^[7] ^and *v*^[8]^. The remaining rows (rows 16 to 20) are Gaussian pseudorandom numbers.

• *D_b _*∈ ℝ^20×50^: the first 5 rows are linear combinations of *v*^[1]^, *v*^[2]^, *v*^[3]^, *v*^[4]^, *v*^[5]^, *v*^[6] ^and *v*^[7]^. Rows 6 to 14 are Gaussian pseudorandom numbers. The remaining rows (rows 15 to 20) are combinations of *v*^[4] ^and *v*^[9]^.

Building up the rows from the underlying signals in this manner allowed us to construct the correlation patterns seen in Figure 4.

**Figure 6:**
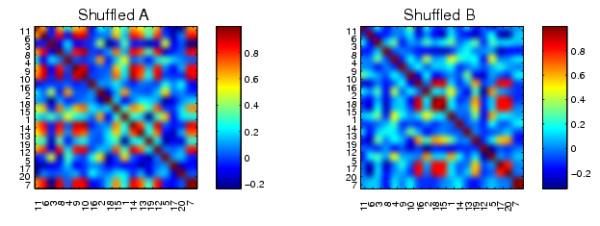
**Relabeled versions of the synthetic networks in Figure 4**.

Although the algorithm is invariant to permutation, for visual clarity, we also shuffled the synthetic correlation data sets *A *and *B *before applying our algorithm to them. Figure 6 shows the same synthetic correlation data sets with an arbitrary relabeling.

**Figure 7:**
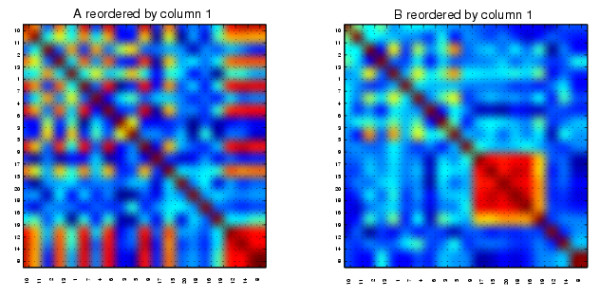
**The synthetic correlation data reordered with the first column from ***X ***^-***T***^**.

**Figure 8:**
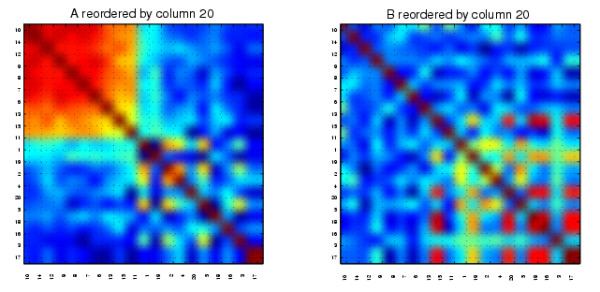
**The synthetic correlation data reordered with the final column from ***X ***^-***T***^**.

We present the results from our algorithm in Figures 7 and 8. We show the relabeled *A *and *B *reordered with two extreme columns of *X *^-*T*^, one from each of the two ends of the spectrum. The reorderings reveal the mutually exclusive cluster structures of *A *and *B*. We also applied the cluster validation method to the structures uncovered by the reorderings using random permutation. In Figure 7 we see that the first column of *X *^-*T *^picks out the continuous nodes 17, 15, 20, 18, 16, 19, which form a good cluster in *B *but not in *A *(*p *< 0.001). The reordering from the final column of *X *^-*T *^shown in Figure 8 reveals that the 6-15 cluster in *A *but not in *B *was completely uncovered by the nodes 10, 14, 12, 9, 8, 7, 6, 13, 15, 11 (at the top left hand side of the heatmaps, *p *< 0.001).

In summary, this additional synthetic test illustrates that our GSVD based algorithm can be extended to reveal the pattern difference between two relative correlation matrices in terms of clustering.

### Quantitative determination of metabolic pathways disrupted in the prefrontal cortex of PCP-treated animals

SIEVE analysis (Thermo-Fisher Scientific) revealed significant PCP-induced alterations in the level of specific metabolites in the PFC of PCP-treated rats (Table 1 Additional File 1). These changes were evident in multiple metabolic pathways as defined by the Kyoto Encyclopedia of Genes and Genomes (KEGG) metabolite pathways database. Significant changes were evident in (i) glutamate metabolism (3 metabolites [m, n]), (ii) the alanine, aspartate and glutamate pathway (2 metabolites [n]), (iii) phenylalanine, tyrosine and tryptophan metabolism (3 metabolites [a]), (iv) purine metabolism (2 metabolites [o]) and (v) butanoate metabolism (2 metabolites [k]). This suggested that these metabolic pathways are disrupted in the PFC of PCP-treated animals. However, this simple level of analysis prevents any quantitative and statistically rigorous determination of the predefined (KEGG) metabolic pathways disrupted in the PFC of PCP-treated animals.

**Table 1:** PCP-induced alterations in PFC metabolite levels as determined by SIEVE analysis

Formula	Metabolite	Metaboite KEGG ID	KEGG Pathways	***P *****-value**	Ratio
*C*_9_*H*_11_*NO*_3_	L-Tyrosine	c00082	ko00350, ko00360, ko00400	0.001	0.584

*C*_10_*H*_17_*N*_3_*O*_6_	gamma Glutamylglutamine	NA	NA	0.007	0.673

*C*_6_*H*_13_*N*_3_*O*_3_	L-Citrulline	c00327	ko00330	0.007	0.709

*C*_3_*H*_7_*NO*_2_*S*	L-Cysteine	c00097	ko00260, ko00270, ko00430, ko00480, ko00730, ko00770, ko00920	0.012	0.445

*C*_8_*H*_9_*NO*	2-Phenylacetamide	c02505	ko00360	0.015	0.561

*C*_9_*H*_8_*O*_3_	Phenylpyruvate	c00166	ko00360, ko00400	0.016	0.57

*C*_4_*H*_6_*O*_2_	2,3-Butanedione	c00741	map00650	0.017	0.786

*C*_4_*H*_5_*N*_3_*O*	Cytosine	c00380	ko00240	0.019	0.665

*C*_04_*H*_9_*NO*_2_	GABA	c00334	ko00250, ko00330, ko00410, ap00650, ko04080	0.021	0.804

*C*_9_*H*_17_*NO*_4_	O-Acetylcarnitine	c02571	ko00250	0.022	2.649

*C*_14_*H*_18_*N*_5_*O*_11_*P*	Adenylosuccinate	c03794	ko00230, ko00250	0.029	3.276

*C*_5_*H*_5_*N*_5_*O*	Guanine	c00242	ko00230	0.035	0.593

*C*_7_*H*_16_*NO*_3_	Carnitine	c00487	ko00310	0.037	0.819

Table 1 shows the molecular formula, tentative metabolite identity and the KEGG pathways in which a metabolite is involved. Only metabolites found to be significantly different between the two experimental groups by SIEVE analysis (see Methods section) are shown. Full data for all metabolites detected in the PFC of control and PCP-treated rats are shown in Table S1 (Additional File 1). The most prominent alterations in KEGG defined metabolic pathways appeared to be in **(i) **alanine, aspartate and glutamate metabolism (3 metabolites [ko00250]), **(ii) **phenylalanine, tyrosine and tryptophan metabolism (3 metabolites [ko00360]), **(iii) **purine metabolism (2 metabolites [ko00230]) and **(iv) **butanoate metabolism (2 metabolites [ko00650]). KEGG defined metabolic pathways; **ko00250**: Alanine, Aspartate and Glutamate metabolism; **ko00330**: Arginine and Proline metabolism; **ko00410**: beta-Alanine metabolism; **map00650**: Butanoate metabolism; **ko00270**: Cysteine and Methionine metabolism; **k00480**: Glutathione metabolism; **ko00260**: Glycine, Serine and Threonine metabolism; **ko00430**: Methionine metabolism; **ko04080**; Neuroactive ligand-receptor interaction; **ko00770**: Pantoate and CoA biosynthesis; **ko00360**: Phenylalanine metabolism; **ko00400**: Phenylalanine, Tyrosine and Tryptophan biosynthesis; **ko00230**: Purine metabolism; **ko00240**: Pyrimidine metabolism; **ko00920**: Sulphur metabolism; **ko00430**: Taurine and Hypotaurine metabolism; **ko00730**: Thiamine metabolism; **ko00350**: Tyrosine metabolism; **ko00400**: Tyrosine and Tryptophan biosynthesis. NA denotes a metabolite not associated with a KEGG compound ID or KEGG pathway.

In the context of this study the aim of applying the GSVD algorithm to metabolomic data from control and PCP-treated animals was to quantitatively determine which predefined metabolic pathways were altered in PCP-treated animals. The inter-metabolite Pearson's correlation coefficient (partial correlation) was used as the metric of the functional association between each pair of metabolites and was generated from the metabolite peak intensities, as determined by Liquid Chromatography Mass Spectrometry (LC-MS), across all animals within the same experimental group (i.e. either control or PCP-treated). These correlations were Fisher transformed to give the correlation data a normal distribution. This resulted in a pair of symmetric, square, real-valued {98 × 98} partial correlation matrices (Control animals: Additional File 2 PCP-treated animals: Additional File 3). Each within-group matrix represents the specific association strength between each of the 9506 possible pairs of metabolites in that experimental group. In the simplest biological case the correlation coefficient between two metabolites (nodes) in the matrix represents the series of enzymatic reactions responsible for converting one metabolite into another. However, it should be noted that this simple interpretation does not account for the complex relationships that may influence the correlation between two metabolites, such as the involvement of metabolites in alternative, often parallel, metabolic pathways. There are important limitations that must be recognized when modeling metabolomic data as a complex network of interactions between metabolites (as defined by the correlation that exists between them) such as the potential for correlations to exist between metabolites that are not biologically relevant. The impact of such erroneous associations on the interpretation of the data as outlined in this paper will be limited by the approach of characterizing alterations at the level of metabolic pathways, involving multiple metabolites (the approach taken in this study), rather than considering the disruption of single correlation coefficient between two metabolites.

**Figure 9:**
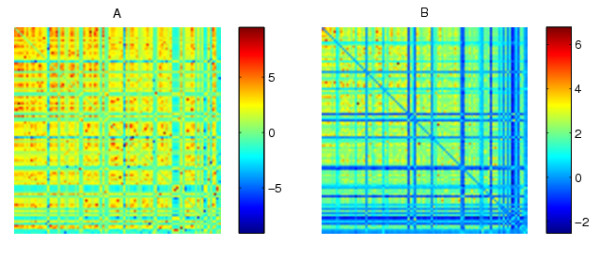
**Control and PCP: original ordering**.

**Figure 10:**
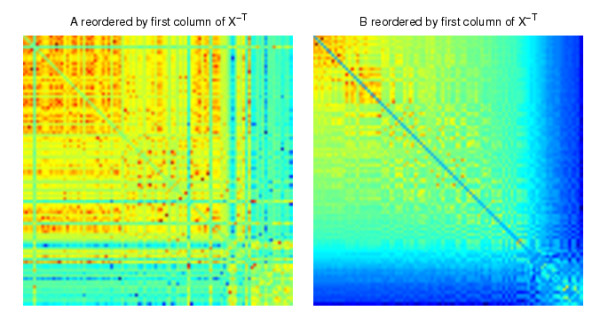
**Control (***A***) and PCP (***B***): reordered with the first column from ***X ***^-***T***^**.

**Figure 11:**
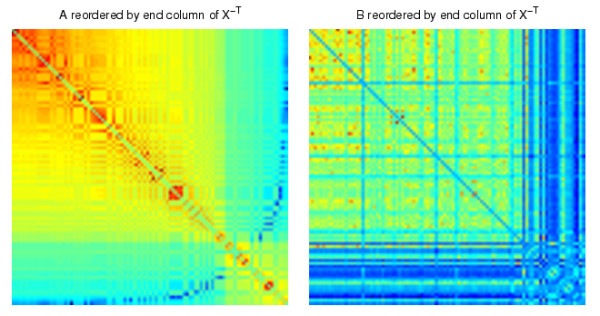
**Control (***A***) and PCP (***B***): reordered with the final column of ***X ***^-***T***^**.

Our network treats interactions between molecules as bidirectional, and so the set of interactions between molecules forms an undirected weighted network. In essence the GSVD algorithm allows the reordering of the two experimental matrices *A *(control animals) and *B *(PCP-treated animals) with the aim of discovering a new node (metabolite) ordering that reveals clusters of nodes that exhibit strong connectivity (mutual weights) in one network but not the other. In the context of this data the GSVD algorithm was used to identify clusters of metabolites present in one experimental group that are not present in the other with the aim of identifying those metabolic pathways in the PFC disrupted by PCP treatment. Once the matrices had been reordered through the GSVD algorithm the significant presence of a cluster in the given network was statistically tested by comparison of the cluster quality measure in the real networks relative to that in 1000 random permutations of the initial matrices. The original metabolomic networks are shown in Figure 9, where matrix *A *represents control animals and *B *represents PCP-treated animals. Figures 10 and 11 show the networks reordered by the first and the final column of *X ^-T^*, respectively. The original position of each metabolite detected by LC-MS (Figure 9) and its new position in each of the reordered matrices (Figures 10 and 11) are shown in Additional File 4. Visually, in Figure 10 there was no obvious pattern of clustering that would identify significant clusters of metabolites present in PCP-treated animals that were not present in controls. In contrast, in Figure 11 there appeared to be clusters of metabolites present in the PFC of control animals that were not present in PCP-treated animals (top left and bottom right hand side of the heatmap). For Figure 11 the significance of the top cluster (first 22 nodes in the reordering, *p *< 0.001) and the bottom cluster (last 18 nodes in the reordering, *p *< 0.001) was confirmed, indicating that there were clusters of metabolites significantly present in control (*A*) animals that were not present in PCP-treated (*B*) animals. The identity of the metabolites, the KEGG pathways in which each metabolite is involved, and the PCP-induced alteration in the overt level of each metabolite (as determined by SIEVE analysis) are shown in Tables 2 and 3 for the top and bottom cluster, respectively. In contrast to the metabolite clustering shown in Figure 11 there was no evidence in Figure 10 for any significant cluster of metabolites present in PCP-treated animals (*B*) that was not present in control (*A*) animals: (i) potential top cluster [first 10 nodes] *p *= 0.421; (ii) potential middle cluster [nodes 18-25] *p *= 0.494.

**Table 2:** Metabolite identities and their relevant KEGG pathways in the top cluster of Figure 11

Formula	Metabolite	Metaboite KEGG ID	KEGG Pathways	*P *-value	Ratio
*C*_5_*H*_10_*N*_2_*O*_3_	L-Glutamine	c00064	Ko00230, ko00240, ko00250, ko00330	0.522	0.959

*H*_3_*PO*_4_	Phosphoric acid	c00009	ko00190	0.254	0.915

*C*_5_*H*_7_*NO*_3_	1-Pyrroline-4-hydroxy-2- carboxylate	c04282	ko00330	0.781	0.981

*C*_4_*H*_9_*N*_3_*O*_2_	Creatine	c00300	ko00330, ko00260	0.551	0.953

**C_4_H_9_NO_2_**	**GABA**	**c00334**	**ko00250, ko00330, ko00410, ko04080, map00650**	**0.021**	**0.804**

*C*_4_*H*_7_*NO*_4_	L-Aspartate	c00049	ko00250, ko00260, ko00270, map00300, ko00330, ko00340, ko00410, ko00760, ko00770, ko04080	0.319	0.916

*C*_4_*H*_7_*NO*_2_	1-Aminocyclopropane-1-carboxylate	c01234	ko00270, ko00640	0.590	0.951

**C_5_H_5_N_5_O**	**Guanine**	**c00242**	**ko00230**	**0.035**	**0.593**

*C*_5_*H*_9_*NO*_4_	Glutamate	c00025	ko00250, ko00330, ko00340, ko00471, ko04080, ko00480, map00650	0.845	0.985

*C*_4_*H*_7_*NO*	Hydroxymethylpropanitrile	NA	NA	0.098	0.842

C_6_*H*_6_*N*_2_*O*	Nicotinamide	c00153	ko00760	0.440	0.917

**C_4_H_6_O_2_**	**2,3-Butanedione**	**c00741**	**map00650**	**0.017**	**0.786**

*C*_6_*H*_12_*O*_4_	Pantoate	c00552	ko00770	0.722	0.963

*C*_15_*H*_23_*N*_5_*O*_14_*P*_2_	ADP-ribose	c00301	ko00230	0.058	677.029

*C*_3_*H*_7_*NO*_3_	L-Serine	c00065	ko00260, ko00270, ko00600, ko00920, ko00680	0.316	0.856

**C_4_H_5_N_3_O**	**Cytosine**	**c00380**	**ko00240**	**0.019**	**0.665**

*C*_2_*H*_7_*NO*_3_*S*	Taurine	c00245	ko00430, ko04080	0.936	0.995

*C*_4_*H*_5_*NO*_3_	Maleamate	c01596	ko00760	0.372	0.927

*C*_2_*H*_8_*NO*_4_*P*	Ethanolamine phosphate	c00346	ko00260, ko00564, ko00600	0.373	0.889

	Unknown ID	NA	NA	0.271	1.395

*C*_5_*H*_11_*NO*_3_	Hydroxyvaline	NA	NA	0.585	0.946

**C_6_H_13_N_3_O_3_**	**L-Citrulline**	**c00327**	**ko00330**	**0.007**	**0.709**

Table 2 shows the top cluster of metabolites identified by the GSVD algorithm that are present in the PFC of control but not PCP-treated animals (Figure 11). The molecular formula, tentative molecular identity, its KEGG compound identity and the KEGG metabolic pathways in which a given metabolite is involved are also shown. The key for each KEGG pathway identity is shown in Table 4. The *p *-values and ratio change reported for each metabolite in this table are those calculated by SIEVE analysis. Those metabolites found to be significantly different between the two groups by analysis are highlighted in bold. While SIEVE analysis fails to attribute significance (*p *< 0.05) to PCP-induced alterations in the overt concentration of many metabolites in this cluster, GSVD analysis reveals that the relationship between these metabolites is significantly altered by PCP treatment (*p *< 0.001), highlighting the specific metabolic pathways that may be disrupted in the PFC of PCP-treated animals. The most prominent alterations in KEGG defined pathways in this cluster were in **(i) **Arginine and Proline metabolism (7 metabolites [ko00330]) **(ii) **Glycine, Serine and Threonine metabolism (3 metabolites [ko00260]) and **(iii) **KEGG defined neuroactive ligands (4 metabolites [ko04080]).

**Table 3:** Metabolite identities and their relevant KEGG pathways in the bottom cluster of Figure 11

Formula	Metabolite	Metaboite KEGG ID	KEGG Pathways	***P *****-value**	Ratio
*C*_5_*H*_4_*N*_4_*O*_2_	Xanthine	c00385	ko00230	0.339	0.508

*C*_10_*H*_16_*N*_2_*O*_7_	Gamma- glutamylglutamic acid	NA	NA	0.143	0.54

*C*_14_*H*_26_*O*_2_	Myristoleic acid	c08322	NA	0.689	0.623

*C*_5_*H*_4_*N*_4_*O*	Hypoxanthine	c00262	ko00230	0.115	0.569

*C*_17_*H*_37_*NO*_2_	Heptadecasphinganine	NA	NA	0.733	0.769

*C*_10_*H*_13_*N*_4_*O*_8_*P*	Inosine monophosphate	c00130	ko00230	0.461	0.73

*C*_10_*H*_17_*N*_3_*O*_6_	Peptide fragment (Arg-Arg-Gln)	NA	NA	0.775	1.183

*C*_6_*H*_15_*NO*_3_	Triethanolamine	c06771	ko00564	0.691	1.207

*C*_9_*H*_14_*N*_4_*O*_3_	Carnosine	c00386	ko00340, ko00410	0.872	1.128

*C*_10_*H*_12_*N*_4_*O*_5_	Inosine	c00294	ko00230	0.090	0.6

*C*_15_*H*_12_*O*_5_	Narigenin	c00509	NA	0.196	0.862

*C*_10_*H*_17_*N*_3_*O*_6_	gamma Glutamylglutamine	NA	NA	0.007	0.673

*C*_26_*H*_42_*N*_7_*O*_20_*P*_3_*S*	2-Hydroxyglutaryl-CoA	c03058	map00650	0.179	0.715

*C*_31_*H*_54_*N*_7_*O*_17_*P*_3_*S*	Decanoyl-CoA	c05274	ko00071	0.410	1.312

*C*_25_*H*_44_*NO*_7_*P*	2- Aminoethylphosphocholate	c05683	ko00440	0.243	0.662

*C*_22_*H*_26_*O*_6_	Eudesmin	NA	NA	0.084	0.493

*C*_3_*H*_7_*NO*_2_*S*	L-Cysteine	c00097	ko00260, ko00270, ko00430, ko00480, ko00730, ko00770, map00920	0.012	0.445

*C*_3_*H*_7_*O*_6_*P *)	Glycerone phosphate	c00111	ko00010, ko00051, ko00052, ko00561, ko00562, ko00564, ko00620	0.063	0.381

Table 3 concerns the bottom cluster of metabolites identified by the GSVD algorithm that are present in the PFC of control animals but not PCP-treated animals. The molecular formula, tentative molecular identity, its KEGG compound identity and the KEGG metabolic pathways in which a given metabolite is involved are shown. The identity of each KEGG pathway ID is shown in Table 5. The *p *-values and ratio change reported for each metabolite in this table are those calculated by SIEVE analysis. Those metabolites found to be significantly different between the two groups by analysis are highlighted in bold. While SIEVE analysis fails to attribute significance (*p *< 0.05) to PCP-induced alterations in the overt concentration of many metabolites for many metabolites in this cluster, the PCP/Control ratio suggests that the levels of many of these metabolites are markedly altered by PCP-treatment. GSVD analysis reveals that the relationship between the levels of these metabolites in this cluster are significantly altered by PCP-treatment (*p *< 0.001) highlighting specific metabolic pathways that may be disrupted in the PFC of PCP-treated animals. There appears to be an overabundance of Purine (4 metabolites [ko00230]) and Glycerophospholipid (2 metabolites [ko00564]) in the bottom cluster.

Rigorous significance testing, involving multiple potential metabolite clusters, confirmed that there were no significant clusters of metabolites in PCP-treated animals that were not present in controls (Figure 10). Following significance testing of potential metabolite clusters in the GSVD reordered matrices, hypergeometric probability (described in the Methods section) was applied to test the significance of KEGG defined metabolite pathway over-representation in these clusters. The results for hypergeometric probability testing are shown in Tables 4 and 5.

**Table 4:** Hypergeometric probability of KEGG defined metabolic pathways in the top cluster in Figure 11

KEGG Path-way Identity	KEGG Pathway	Number of metabolites in cluster(A)	Total number of pathway metabolites detected (B)	Hypergeometric Probability (*P *(*X*) ≥ *k*)
ko00190	Oxidative phosphorylation	1	1	0.224

ko00230	Purine metabolism	3	13	0.598

ko00240	Pyrimidine metabolism	2	6	0.406

**ko00250**	**Alanine, Aspartate and Glutamate metabolism**	**4**	**7**	**0.043**

**ko00260**	**Glycine, Serine and** **Threonine metabolism**	**4**	**7**	**0.043**

ko00270	Cysteine and Methionine metabolism	3	7	0.186

map00300	Lysine biosynthesis	1	3	0.538

**ko00330**	**Arginine and Proline metabolism**	**7**	**10**	**0.001**

ko00340	Histidine metabolism	2	5	0.312

ko00410	beta-Alanine metabolism	2	5	0.312

ko00430	Taurine and Hypotaurine metabolism	1	3	0.538

ko00471	D-glutamine and D-glutamate metabolism	1	1	0.224

ko00480	Glutathione metabolism	1	5	0.728

ko00564	Glycerophospholipid metabolism	1	11	0.949

ko00600	Sphingolipid metabolism	2	3	0.126

ko00640	Propanoate metabolism	1	2	0.400

**map00650**	**Butanoate metabolism**	**3**	**4**	**0.034**

ko00680	Methane metabolism	1	1	0.224

**ko00760**	**Nicotinate and Nicotinamide metabolism**	**3**	**4**	**0.034**

ko00770	Pantothenate and CoA biosynthesis	2	5	0.312

ko00920	Sulphur metabolism	1	3	0.538

**ko04080**	**Neuroactive ligand-receptor interaction**	**4**	**7**	**0.043**

Table 4 shows the hypergeometric probability of at least the observed number of metabolites arising by chance for a given KEGG defined metabolic pathway in the top cluster of Figure 11, identified through the GSVD algorithm as being present in control but not PCP-treated animals. Further computational details are given in the Methods section. The cluster size was 22 metabolites from a total population of 98. There was a significant over representation of metabolites of **(i) **Alanine, Aspartate and Glutamate metabolism [ko00250], **(ii) **Arginine and Proline metabolism [ko00330], **(iii) **Butanoate metabolism [ko00650], **(iv) **Nicotinate and Nicotinamide metabolism [ko00760], **(v) **Glycine, Serine and Threonine metabolism and **(vi) **those metabolites active as neurotransmitter ligands [ko04080] (all highlighted in bold) suggesting that these pathways are disrupted in the PFC of PCP-treated animals.

**Table 5:** Hypergeometric probability of KEGG defined metabolic pathways in bottom cluster in Figure 11

KEGG Path-way Identity	KEGG Pathway	Number of metabolites in cluster(A)	Total number of pathway metabolites detected (B)	Hypergeometric Probability (*P *(*X*) ≥ *k*)
ko00010	Glycolysis/Gluconeogenesis	1	1	0.184

ko00051	Fructose and Mannose metabolism	1	1	0.184

ko00052	Galactose metabolism	1	1	0.184

ko00071	Fatty acid metabolism	1	1	0.184

ko00230	Purine metabolism	4	13	0.191

ko00260	Glycine, Serine and Threonine metabolism	1	7	0.770

ko00270	Cysteine and Methionine metabolism	1	7	0.770

ko00340	Histidine metabolism	1	5	0.646

ko00410	beta-Alanine metabolism	1	5	0.646

ko00430	Taurine and Hypotaurine metabolism	1	3	0.460

ko00440	Phosphonate and Phosphinate metabolism	1	2	0.335

ko00480	Glutathione metabolism	1	5	0.646

ko00561	Glycerolipid metabolism	1	2	0.335

ko00562	Inositol Phosphate metabolism	1	2	0.335

ko00564	Glycerphopholipid metabolism	2	11	0.642

ko00620	Pyruvate metabolism	1	2	0.335

map00650	Butanoate metabolism	1	4	0.562

ko00730	Thiamine metabolism	1	1	0.184

ko00770	Pantothenate and CoA biosynthesis	1	5	0.646

map00920	Sulphur metabolism	1	3	0.460

Table 5 shows the hypergeometric probability of randomly seeing at least the observed number of metabolites of a given KEGG pathway in the bottom cluster of Figure 11, identified though the GSVD algorithm as being present in control animals but not in PCP-treated animals. There was no evidence for a particular over-abundance of metabolites from any given KEGG pathway in this cluster. Cluster size is 18 metabolites from a total population of 98.

## Discussion

Through its application to metabolomic data we have clearly demonstrated the added value that can be gained from applying the GSVD algorithm to two sets of complex, network data based upon the same set of nodes. In particular, we have demonstrated that the combined application of the GSVD algorithm with hypergeometric probability analysis provides an analytical framework by which statistical alterations in predefined metabolic pathways between experimental groups can be defined from complex metabolomic data. There is a great unmet need for this type of analytical approach in metabolomics, as well as in the other -omics fields (e.g. transcriptomics), which allows the quantification of alterations at the biological systems (pathways) level rather than simply identifying significant alterations of discrete measures (i.e. single metabolites).

Through the application of this analytical approach we identified statistically significant alterations in specific, pre-defined metabolic pathways (KEGG database pathways) that may contribute to PFC dysfunction in PCP-treated animals, and so in schizophrenia. This included the disruption of the (1) Alanine, Aspartate and Glutamate [ko00250], (2) Arginine and Proline [ko00330], (3) Butanoate [ko00650], (4) Nicotinate and Nicotinamide [ko00760], (5) Glycine, Serine and Threonine metabolic pathways as well as an imbalance in (6) metabolites active as neurotransmitter ligands [ko04080]. The disruption of metabolic pathways involving glutamate in the PFC of PCP-treated rats seems particularly pertinent given the reported alterations in extracellular glutamate availability in the PFC following repeated PCP treatment [53] and the central hypothesis of hypofunctional glutamatergic PFC neurotransmission in schizophrenia [54,55]. In addition to altered glutamate metabolism there was also evidence to support an imbalance in multiple metabolites known to be active at glutamate receptors. This included an imbalance in the relationship between glutamate, L-aspartate and Tauring (Table 2) which are all known to be active at glutamate receptors. Furthermore, evidence for the disruption of glycine, serine and threonine metabolism may suggest that glycine and serine activity as co-agonists at the NMDA receptors may be disrupted in the PFC of PCP-treated animals. However, it is important to note that we failed to detect glycine levels in our samples and serine levels appear to be overtly unchanged. The possibility of altered glycine levels in the PFC of PCP-treated rats warrants further investigation given the ability of glycine and NMDA receptor glycine site agonists to reverse subchronic PCP-induced alterations in PFC dopaminergic neurotransmission [56,57], which may be central to the impact of subchronic PCP treatment on cognition. Altered glycine, serine and threonine metabolism in the PFC of PCP-treated animals is also consistent with the hypothesis that glycine and serine represent potential therapeutic targets for the treatment of schizophrenia [58]. In addition, we found evidence to suggest that GABA neurotransmission was also significantly decreased in the PFC of PCP-treated rats, which may relate to the compromised integrity of GABAergic interneurones in these animals [3,6], which closely resemble the GABAergic interneuron alterations seen in schizophrenia. The imbalance in glutamate, glutamine and GABA levels identified in the PFC of PCP-treated rats may directly contribute to the hypofrontality (glucose hypometabolism) seen in these animals, as detected using the ^14^C-2-deoxyglucose imaging technique [4], as all of these metabolites are intimately linked through metabolic pathways and have a central role in regulating the coupling of neuronal activity to cerebral glucose metabolism [59,60].

Our results also suggest that glutamatergic dysfunction in the PFC of PCP-treated rats is not limited to the disruption of glutamatergic neurotransmission but also involves the disruption of the metabolic pathways in which glutatmate is involved. For example, altered glutamate metabolism may directly contribute to the disruption of the Arginine-Proline metabolic pathway in the PFC of PCP-treated animals. The significant disruption of the Arginine pathway in PCP-treated animals suggests that prolonged NMDA receptor hypofunction may result in disrupted nitric oxide (NO) signalling in the PFC. There is increasing evidence that NO signalling is directly linked to NMDA receptor activity through regulation of the enzyme nitric oxide synthase (NOS) [61] and that NO signaling contributes to the deficits in cognition that arise from acute NMDA receptor blockade [62,63]. The finding that Citrulline levels, a metabolite in the Arginine-Proline pathway, are significantly decreased in the PFC of the PCP-treated rats in this study further supports the suggestion that NOS activity is altered in the PFC of these animals, as this metabolite is formed by NOS when it releases NO from L-arginine. This suggests that NMDA receptor hypofunction may underlie the decreased NOS activity and protein expression levels reported in the PFC of schizophrenia patients [64] and may contribute to the cognitive deficits seen in this disorder.

In addition to quantitatively defining the specific metabolic pathways altered by experimental manipulation, our results suggest that the GSVD algorithm can identify discrete series of metabolic reactions altered by experimental manipulation. In this way, while we found no significant evidence to support the widespread disruption of purine metabolism, or the significant disruption of any other KEGG defined metabolic pathway in the bottom cluster as detected using the GSVD, we did find evidence in this cluster to suggest that a specific series of purine reactions were significantly disrupted in the PFC of PCP-treated animals. These disrupted purinergic reactions in the PFC of PCP-treated animals were:

This result suggests that the activity of adenylosuccinate synthase (ADSS), the enzyme responsible for the conversion of IMP to adenylosuccinate, may be significantly increased in the PFC of PCP-treated animals. An increase in the functional activity of this enzyme could result in both the increased level of adenylosuccinate and the altered balance in the enzyme's downstream metabolites (IMP, Inosine, Hypoxanthine, Xanthine) seen in the PFC of PCP-treated animals. While the influence of prolonged NMDA receptor hypofunction on the functional activity of this specific enzyme remains to be confirmed, and clearly warrants further systematic investigation, the recent finding of altered ADSS gene expression in schizophrenia [65] and the association of ADSS gene polymorphisms with schizophrenia [66] further highlights a potential role for this metabolic pathway in this disorder. In addition, a role for this metabolic pathway in cognition and schizophrenia is supported by the observation that inherited deficiency in the enzyme responsible for the breakdown of adenylosuccinate (ASL) results in mental retardation and autistic features [67,68]. Furthermore, the ASL gene maps to chromosome 22*q*13.1-*q*13.2 in humans [69] and these chromosomal loci have been repeatedly linked to schizophrenia [70-72]. The disruption of this metabolic pathway may also contribute to the reduced rate of cerebral glucose metabolism in the PFC of PCP-treated animals [3,4] as ASL deficiency results in hypometabolism in frontal cortical structures [73]. Overall, these results suggest that the potential role of this specific series of metabolic reactions and its enzymes in cognition and schizophrenia warrants further investigation.

## Conclusions

This work addresses the scenario where a pair of networks describes two different patterns of connection between a common set of nodes. We argued from first principles that the Generalized Singular Value Decomposition (equation (4)) can form the basis of a very useful computational tool. In practice, we have shown that this new computational network reordering technique was able to identify alterations in metabolic pathways in the PFC of rats treated subchronically with PCP that may contribute to the PFC dysfunction and cognitive deficits seen in these animals. Furthermore, the metabolic pathways identified as being disrupted in the PFC of PCP-treated rats trough the application of this new computation technique clearly overlap with those metabolic species known to be disrupted in schizophrenia. Applying this new algorithm in this way also identified novel pathways that may also be relevant to schizophrenia. In this way we identified alterations in glutamate metabolism and metabolic pathways central to glutamatergic neurotransmission, alterations in arginine and proline metabolism and the disruption of a novel series of purine reactions that may contribute to the PFC dysfunction and cognitive deficits seen in schizophrenia.

## Methods

### Chemicals

The solvents used for the study were purchased from the following sources: Acetonitrile, methanol and chloroform (Fisher Scientific, Leicestershire, UK) and formic acid (VWR, Poole, UK). All chemicals used were of analytical reagent grade. A Direct Q-3^® ^water purification system (Millipore, Watford, UK) was used to produce HPLC grade water which was used in all analysis. Standards for 90 common bio-molecules were also purchased which were used to characterize the ZIC-HILIC column (Sigma Aldrich, Dorset UK).

### Animals

All experiments were completed using male Lister Hooded rats (Harlan-Olac, UK) housed under standard conditions (21°C, 45-65% humidity, 12-*h *dark/light cycle (lights on 0600*h*) with food and drinking water available *ad libitum*). All manipulations were carried out at least 1 week after entry into the facility and all experiments were carried out under the Animals (Scientific Procedures) Act 1986. Animals received either sub-chronic treatment with vehicle (0.9% saline, *i.p*., *n *= 5) or 2.58*mg.kg*^-1 ^PCP.HCl (*i.p*., Sigma Aldrich, UK) once daily for five consecutive days (*n *= 5). At 72 hours after the final drug treatment dose animals were sacrificed and the brain rapidly dissected out and frozen in isopentane (-40°C) and stored at -80°C until sectioning. Frozen brains were sectioned (20 *μM*) in the coronal plane in a cryostat (-20°C). Tissue sections from the prefrontal cortex (PFC, Bregma +4.70*mm *to Bregma +3.20*mm*) were collected in 4*ml *glass vials with reference to a stereotactic rat brain atlas [74] and stored at -80°C until further preparation for LC-MS analysis.

### Extraction of polar metabolites from brain samples for LC-MS analysis

Extraction of polar metabolites from brain tissue was carried out using the two-step extraction method described previously [75], using methanol, water and chloroform for the optimal extraction of polar metabolites. A hand held homogenizer was used to homogenize the samples once in solution. For preparation of samples for LC-MS analysis 200 *μl *of the collected polar extract was added to 600 *μl *of 1 : 1 acetonitrile:water solution to produce a final solvent:sample ratio of 3 : 1. The samples were then filtered using Acrodisc 13*mm *syringe filters with 0.2 *μm *nylon membrane (Sigma Aldrich) before LC-MS analysis.

### LC-MS analysis of polar metabolites

Experiments were carried out using a Finnigan LTQ Orbitrap (Thermo Fisher, Hemel Hempstead, UK) using 30000 resolution. Analysis was carried out in positive mode over a mass range of 60-1000 *m*/*z*. The capillary temperature was set at 250°C and in positive ionization mode the ion spray voltage was 4.5 *kV *, the capillary voltage 30 *V *and the tube lens voltage 105 *V *. The sheath and auxiliary gas flow rates were 45 and 15, respectively (units not specified by manufacturer). A ZIC-HILIC column (5 *μm*, 150 × 4.6 *mm*; HiChrom, Reading, UK) was used in all analysis and a binary gradient method was developed which produced good polar metabolite separation. Solvent A was 0.1% *v*/*v *formic acid in HPLC grade water and solvent B was 0.1% *v*/*v *formic acid in acetonitrile. A flow rate of 0.3 *ml*/*min*. was used and the injection volume was 10 *μl*. The gradient programme used was 80% B at 0 *min*. to 50% B at 12 *min*. to 20% B at 28 *min*. to 80% B at 37 *min*., with total run time of 45 minutes. The instrument was externally calibrated before analysis and internally calibrated using lock masses at *m*/*z *83.06037 and *m*/*z *195.08625. Samples were analysed sequentially and the vial tray temperature was set at a constant temperature of 4°C.

### Data preparation and analysis

#### Determination of overt alterations in metabolite levels between experimental groups

The software program Xcalibur (version 2.0) was used to acquire the LC-MS data. The raw Xcalibur data files from version 1.2 (Thermo Fisher, Hemel Hempstead, UK). SIEVE software (Thermo-Fisher Scientific) was used to identify all metabolites affected by drug treatment by calculating a *p*-value and ratio based on the difference in average intensities of individual peaks, which correspond to different metabolites, between PCP-treated and control animals. A significant difference in the level of each metabolite between groups was set at *p *-value < 0.05 and/or ratio less than 0.5 for downregulated metabolites and greater than 2 for upregulated metabolites. The ratio is the fold change in average peak intensities from control and treatment groups. For metabolite identification the masses of the polar metabolites were compared to the exact masses of 6000 biomolecules using an in-house developed macro (Excel, Microsoft 2007).

#### Hypergeometric probability testing

The hypergeometric probability test was used to calculate the probability of finding at least the observed number of metabolites of a given pre-defined metabolic pathway (as defined on the KEGG pathway database) in the clusters identified through the GSVD algorithm, with knowledge of the total number of metabolites present in that pathway detected by LC-MS in these samples. The hypergeometric probability test was used to identify whether any of the KEGG defined metabolic pathways were significantly over-represented in any of the GSVD identified clusters. In its general form hypergeometric probability allows the calculation of the probability of observing at least (*k*) metabolites from a given defined KEGG pathway in a defined cluster of metabolites (*n*) given the total number of metabolites (*N*) and the total number of metabolites from the pathway in question (*m*). The probability mass function of hypergeometric distribution is:(9)

So here the probability is calculated using the formula(10)

Significant over-representation of a given functional group in any GSVD defined significant cluster was set by a hypergeometric probability threshold of 0.05.

## Authors' contributions

All authors contributed extensively to the work presented in this paper. DJH and XX conceived and analyzed the computational algorithm, designed and performed the synthetic tests and wrote the description of this material. XX applied the algorithm to metabolic networks. ND performed the *in vivo *experiments, analyzed the metabolomic data and wrote the description of this material. BJM and JAP conceived the subchronic PCP model. LM and DGW conducted the metabolomics experiments. All authors discussed the results, interpreted the data and have read and approved the final version of the manuscript.

## Supplementary Material

Additional file 1 **Table S1 - List of all metabolites detected by LC-MS in the PFC of Control and PCP-treated animals**. Table S1 Legend: The molecular formula and tentative molecular identity for each metabolite detected in the PFC of control and PCP-treated animals is shown. In addition, the KEGG molecular identity and the KEGG metabolic pathways in which a metabolite is involved are also shown. The ratio difference in metabolite concentration and the significance of this change (p-value), as determined by SIEVE analysis (see Methods section), are also shown. Those metabolites found to be significantly different between the two groups are highlighted in bold. The most prominent alterations in KEGG defined metabolic pathways appeared to be in **(i) **alanine, aspartate and glutamate metabolism (3 metabolites [ko00250]), **(ii) **phenylalanine, tyrosine and tryptophan metabolism (3 metabolites [ko00360]), **(iii) **purine metabolism (2 metabolites [ko00230]) and **(iv) **butanoate metabolism (2 metabolites [ko00650]). KEGG defined metabolic pathways; **ko00250**: Alanine, Aspartate and Glutamate metabolism; **ko00627**: Aminobenzoate degradation; **ko00330**: Arginine and Proline metabolism; **ko00410**: beta-Alanine metabolism; **ko00780**: Biotin metabolism; **map00650**: Butanoate metabolism; **ko04973**: Carbohydrate metabolism; **ko00270**: Cysteine and Methionine metabolism; **ko00071**: Fatty acid metabolism; **ko00051**: Fructose and Manose metabolism; **ko00052**: Galactose metabolism; **ko00471**: Glutamine and Glutamate metabolism; **k00480**: Glutathione metabolism; **ko00561**: Glycerolipid metabolism; **ko00564**: Glycerophospholipid metabolism; **ko00260**: Glycine, Serine and Threonine metabolism; **ko00010**: Glycolysis/Gluconeogenesis; **ko00340**: Histidine metabolism; **ko00562**: Inositol Phosphate metabolism; **map00300**: Lysine biosynthesis; **ko00310**: Lysine degradation; **ko00430**: Methionine metabolism; **ko04080**; Neuroactive ligand-receptor interaction; **ko00760**: Nicotinate and Nicotinamide metabolism; **ko00190**: Oxidative phosphorylation; **ko00770**: Pantoate and CoA biosynthesis; **ko00550**: Peptidoglycan biosynthesis; **ko00360**: Phenylalanine metabolism; **ko00400**: Phenylalanine, Tyrosine and Tryptophan biosynthesis; **ko00440**: Phosphonate and Phosphinate metabolism; **ko00640**: Propanoate metabolism; **ko00230**: Purine metabolism; **ko00240**: Pyrimidine metabolism; **ko00620**: Pyruvate metabolism; **ko00500**: Starch and Sucrose metabolism; **ko00600**: Sphingolipid metabolism; **ko00920**: Sulphur metabolism; **ko00430**: Taurine and Hypotaurine metabolism; **ko00730**: Thiamine metabolism; **ko00380**: Tryptophan metabolism; **ko00350**: Tyrosine metabolism; **ko00400**: Tyrosine and Tryptophan biosynthesis; **ko00290**; Valine, Leucine and Isoleucine biosynthesis; **ko00280**: Valine, Leucine and Isoleucine degradation. NA denotes a metabolite not associated with a KEGG compound ID or KEGG pathway. Click here for file

Additional file 2 **98 × 98 matrix of between metabolite correlations in the PFC of control animals**. The 98 × 98 matrix of the Pearson's correlation coefficients (Fisher z-transformed) between all metabolites detected in the prefrontal cortex of control (saline-treated) animals by LC-MS analysis is shown. Click here for file

Additional file 3 **98 × 98 matrix of between metabolite correlations in the PFC of PCP-treated animals**. The 98 × 98 matrix of the Pearson's correlation coefficient (Fisher z-transformed) between all metabolites detected in the prefrontal cortex of PCP-treated animals by LC-MS analysis is shown. Click here for file

Additional file 4 **Table S2 - Table showing the axes labels in Figures **9, 10**and **11. In Table S2 the position of each metabolite in the original ordering (Figure 4) is shown. In the columns for Figures 5 and 6, the corresponding numbers indicating the new position of each metabolite (node) in the matrix when reordered by the first column of *X *^-*T *^and the final column of *X *^-*T*^, respectively, is shown. Click here for file
